# Pivots and pirouettes: adapting a robust departmental CPD and training program to the COVID‐19 crisis

**DOI:** 10.1002/jmrs.443

**Published:** 2020-10-18

**Authors:** Jacqueline Besson, Clare McNamara, Elizabeth Brown

**Affiliations:** ^1^ Radiation Oncology Department Princess Alexandra Hospital Woolloongabba Queensland Australia

## Abstract

The onset of the COVID‐19 pandemic caused swift change in society, affecting both personal and professional lives. In radiation therapy (RT), professional and social interactions are highly important to maintaining team culture and effective patient care. Continuing Professional Development (CPD) is also an integral part of maintaining professional and personal competence and growth for healthcare professionals. This article examines the rationale for and methods of swiftly adapting a robust CPD program and training calendar to an online offering for radiation therapists (RTs) at the Princess Alexandra Hospital Radiation Oncology Department, Brisbane, Australia. Reasons for the change, how it was achieved quickly, and the opportunity to build resilience in the staff group are discussed. Successes and challenges of achieving meaningful change in a short timeframe are described, ensuring RTs maintained access to both CPD and social support during the crisis. Initial feedback suggested a positive response from RTs, but the situation remains dynamic and will need to be monitored and adapted as the pandemic continues.

## Graphical abstract

This article examines the rationale for and methods of swiftly adapting our robust CPD program and training calendar to an online offering for RTs at the Princess Alexandra Hospital Radiation Oncology department in Brisbane, Australia. We discuss why such a change was deemed necessary, how we achieved it quickly, and the opportunity to build resilience in our staff group. We address the successes and challenges of achieving meaningful change in a short timeframe, ensuring our RTs maintain access to both CPD and social support during this crisis.

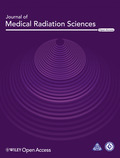

## Introduction

For health professionals, Continuing Professional Development (CPD) is an integral part of maintaining competence and currency of knowledge, playing a role in ensuring that registered practitioners have recent skills, knowledge and attributes for safe, contemporary practice.^1^ Shared CPD activities help staff to broaden horizons and formulate and adhere to personal and professional standards. Health professionals are also used to operating in large multidisciplinary teams, communicating effectively and meaningfully with other team members to ensure a gold standard of patient care is maintained. Radiation therapists (RTs) particularly are accustomed to working within teams that are in constant communication and partaking in shared CPD meetings and activities regularly. The COVID‐19 pandemic meant that many typical interactions at work were stopped almost immediately.

The Princess Alexandra Hospital Radiation Oncology Department has 67 Full Time Equivalent (FTE) RTs. As a large metropolitan tertiary teaching hospital, the department has an RT Education team, consisting of two RTs rostered to Education fulltime for set periods of time from a rotating team of seven educators. A key expectation of the team is to devise and maintain a robust CPD and training program for RT staff.

Normally, this comprises of a varied program each week that includes Peer‐Reviewed Chart Rounds, In‐service sessions, mandatory or practical training coverage, research updates and team meetings. If all sessions were attended an RT could reasonably expect 3–4 h of learning, interaction and engagement provided per week. Our annual calendar is typically filled with invited speakers, booked training sessions and regular updates from staff.

## Situation – Addressing COVID‐19 Changes

In early 2020, the COVID‐19 pandemic drastically changed normal operations, and as such, the Education team was required to adapt quickly to ensure continued offerings of CPD and training opportunities for staff. The changes adopted needed to mitigate the effects of the crisis on the department; including all RT staff isolating into small teams confined to one area and one shift, the loss of meeting spaces and associated audio visual (AV) and computer equipment, re‐deployment of the Education team laptop to frontline areas, and selected Planning, Quality Assurance (QA) and Research staff working from home. The operational changes meant that staff had to stay in their designated areas, and they did not have the opportunity to interact socially during break times as would be usual practice.

Management and the Education team liaised to quickly adapt what was a full and complete activity calendar for 2020 to an online offering, with both educational and social aspects covered. The aim was for staff to be able to digest this content in an hour so that shift overlap time could be utilised.

## Rationale for Approach

The education team remained cognisant of the fact that staff were being asked to cope with an uncertain time both as frontline health professionals and in their personal lives. Healthcare workers reported distress early in the COVID‐19 pandemic, related to the effects of social distancing versus family commitments, and the possibility of illness. Additionally, some staff were also managing underlying health conditions, early pregnancy or caring for others with challenging needs. Disruptions to work life balance can lead to emotional and physical burnout which can increase the risk of errors and infection.^2^

The literature also reports that healthcare workers can often deprioritise their own health and well‐being in favour of patient care, and employers are encouraged to promote self‐care strategies.^3^ Research suggests that staff look to their leaders to provide consistent strategies for well‐being and psychosocial support.^3^ A recent review reported that during the Coronavirus crisis, people need clear and adequate information, and appreciate advice about meaningful activities to reduce psychological impacts while in quarantine.^4^ A regular communication channel should be established by leaders to offer support to staff experiencing significant additional stressors.^2^

Additionally, staff deemed non‐essential who were asked to stay home indefinitely report they felt isolated and ineffective in contributing meaningfully to the crisis and indicated that it was psychologically more satisfying to work than to stay home.^5^

A pandemic may also provide an opportunity to promote resilience and acceptance of change. Traumatic and stressful events can have acute negative effects but can lead to longer term positive outcomes. Healthcare professionals who are supported during difficult times can foster growth from trauma or stressful circumstances.^6^

RTs pride themselves on being adaptable team workers, with all areas of the department always striving to remain connected. An aim at the onset of the crisis was to remain unified as a department and not further isolated. From this perspective staff needed to be supported, within departmental capacity and scope of practice, with both educational and social support that was engaging and focussed on the long‐term morale of the team.

## Our Response

A study of the literature revealed that healthcare professionals seeking online content during a crisis desired interactive and engaging content that can easily be accessed from any device at any time. They prefer collated and packaged material with embedded links or media, enabling easy access to a ‘dip‐in, dip‐out’ approach. The technology employed should be easy to use and readily available to all.^3^

This approach was quickly achieved in the department by adopting a 3‐pronged strategy using Microsoft (MS) products: Teams, Sway and Forms. Scheduled meetings were held via MS Teams, the only meeting software that Queensland Health deemed to meet their security and confidentiality standards. A weekly Educational newsletter and fortnightly social newsletter were distributed using MS Sway for content. Staff were challenged on their knowledge of the content presented in each offering by quizzes utilising MS Forms.

### Educational

MS Teams were utilised for all scheduled doctor peer‐reviewed Chart Rounds, and product demonstrations were held via Teams.

The weekly Educational newsletter, entitled CPD@PA, contained four main sections: an introductory section, an in‐service section, a mindfulness and resilience section and a quiz and reflection section.

The introductory section contained departmental reminders, diary dates and notes, as would be usually addressed at meetings and disseminated through meeting minutes.

The second major section was an in‐service portion, which comprised recorded PowerPoint presentations or videos from our own staff, recordings of MS Teams meetings with product vendors, or replays of publicly available webinars from suppliers. This section often contained a link to a recent journal article, covering topical subjects such as COVID‐19 in Radiation Oncology. One advantage of recording these staff and vendor presentations was that they could be stored for future use in a departmental resource library as they can be replayed or adapted when new technology is implemented.

Our third section comprised a mindfulness and resilience portion, where ideas for staying grounded and mentally healthy in an uncertain world were presented. Examples included guided meditations, relaxation techniques, wellness podcasts and suggestions for kindness and gratitude activities. Staff were understandably experiencing more stress than usual which is why it was important to include a wellness section. These smaller vignettes were designed so that they could be viewed multiple times or shelved and re‐visited at a more appropriate time, such as off site and out of hours.

The newsletter usually finished with a quiz on the content of the in‐service presentation, and some prompts for reflection on the content of the newsletter. Staff were also reminded to self‐record CPD time at the end of the newsletter. Pre‐COVID, CPD points for staff were tracked through a Radio‐frequency identification (RFID) check in system using ID badges; however, this has been replaced by a self‐regulated CPD spreadsheet on the shared drive, accessible to all, including those RTs working from home. It should be noted that engagement with the newsletter and its content was entirely voluntary, though obviously recommended to staff to maintain CPD commitments.

The Education team introduced staff quizzes in 2019 in the hope to improve teamwork skills, bolster camaraderie and to provide some light‐hearted competition between teams. These had been well received and proved quite popular, though are time consuming to produce. When COVID‐19 guidelines were introduced to our department, the Treatment Supervisor sought additional material for RTs to remain engaged in the overlap time between now separated shifts on linacs. Thus, weekly brain teasers were re‐introduced, emailing entertaining and challenging quizzes to all RTs each Wednesday.

While the quiz content was a mixture of educational (e.g. physics and radiation themed puzzles), and social, (e.g. guessing where staff were born, Family Feud style questions, or departmental fact quizzes), the social quizzes were found to be the most popular. These brought out a competitive streak and had the unexpected effect of bringing teams together to answer the questions, involving all shifts and those working from home. This unintended consequence saw a playful and joyous attitude return to the department in what was a stressful time for all. RT work took priority and social distancing was maintained, but the team cohesion was a positive side effect of the quizzes.

### Social

In addition to educational targeted offerings, a fortnightly social newsletter entitled ‘Coffee Break’ was also initiated utilising MS Sway. This was intended as a replacement for tearoom interactions, as it comprised news, events, recipes, reviews and quiz sections. The intention was for the material to take about 20 min to peruse over a cup of tea. The content was light and aimed to foster camaraderie, including items covering department events such as photograph stories of staff on charity dress up days, new pets, engagement announcements, baby showers and birthdays. This provided a way to connect staff in all areas during isolation, especially staff working from home.

Input was received from staff who contributed photographs, answered interview questions, made music, TV, movie and podcast recommendations and suggested articles, links and quizzes. An origami competition and quizzes were held where staff guessed whose pets, plants and holiday snaps were featured. Also included were music, science, sport, history, virtual tours and culture snippets.

## Successes and Challenges

Due to the changing nature of the ongoing crisis, informal feedback only has been gathered at this point. It is intended that a formal analysis and evaluation of the changes will be undertaken in the future. To date, feedback on the new approach has been overwhelmingly positive. Staff have been asked at three different points for their feedback, both via email and through MS Forms. Staff offered suggestions of topics they would like to see covered, training videos they would find useful and teams they would like to see presenting a recorded in‐service session.

An advantage of using MS Sway and Forms applications for newsletters and quizzes is that they could be used to monitor staff engagement, recording how many staff opened each section of the newsletter and how many minutes were spent reading each section. Informal tracking and analysis of Sway engagement over time suggested that most staff were spending appropriate and expected amounts of time perusing each individual section of newsletters, reflecting that the content was topical and suitable. This analysis, along with requested feedback received via email, was taken as a sign of success.

Initial reports suggested some difficulty adapting to Teams meetings, with camera, microphone and connectivity issues. Conversely, some staff reported positive impacts of holding team and peer‐reviewed meetings over Teams, reporting a greater ability to see and hear presentations during meetings.

Sourcing content and constructing the newsletters was time consuming for the Education team, though this was balanced by the postponement of student clinical placements and reduced face‐to‐face student contact compared to prior placements. When student placements and other usual Education Team activities resumed, we distributed the newsletters less often or reduced the amount of content.

It is important to note that engagement and participation in all above activities were voluntary. There was no expectation that staff would contribute to any newsletter or meeting. In no way did the Education team expect to replace usual daily social interactions and a full calendar of activities with only online content. Neither are they trained mental health professionals who can support staff with in‐depth needs, they can only offer and suggest resources. It was not the intention to overwhelm staff with more commitments or information during a challenging time, nor replace qualified psychological professionals.

## Conclusion

Overall, staff adapted quickly to the changes that were forced upon them by circumstance. Engagement and feedback will continue to be monitored and incorporated to ensure quality content for staff. As this crisis continues, it is not yet foreseen when physical meetings and standard CPD calendar activities will recommence. As such, it was reinforced to staff that this is the new normal with CPD and training activities being provided in this manner for the foreseeable future, and that staff themselves will need to be responsible for participating and recording their involvement. Resumption of usual practices of interactive CPD events will be welcomed; however, these online experiences have added a resilient element to the program that can be adapted for future needs.
